# Costs of the COVID-19 vaccination programme: estimates from the West Rand district of South Africa, 2021/2022

**DOI:** 10.1186/s12913-024-11251-1

**Published:** 2024-07-29

**Authors:** Ijeoma Edoka, Lineo Marie Matsela, Khumo Modiba, Yolandie Luther, Sharlene Govender, Thapelo Maotoe, Heena Brahmbhatt, Pedro T. Pisa, Gesine Meyer-Rath, Jacqui Miot

**Affiliations:** 1 Health Economics and Epidemiology Research Office (HE2RO), Faculty of Health Sciences, University of the Witwatersrand, Johannesburg, South Africa; 2 School of Public Health, Faculty of Health Sciences, University of the Witwatersrand, Johannesburg, South Africa; 3 Gauteng Department of Health, West Rand District Health Services, Krugersdorp, South Africa; 4 Right to Care, Pretoria, South Africa; 5 United States Agency for International Development, Pretoria, South Africa; 6 Johns Hopkins Bloomberg School of Public Health, Baltimore, USA; 7 Department of Nutrition, University of Pretoria, Pretoria, South Africa; 8 Department of Global Health, School of Public Health, Boston University, Boston, USA

**Keywords:** COVID-19 vaccination programme, COVID-19 vaccines, Costing analysis, Immunisation economics, Budgeting and planning

## Abstract

**Background:**

The COVID-19 vaccination programme in South Africa was rolled out in February 2021 via five delivery channels- hospitals, primary healthcare (PHC), fixed, temporary, and mobile outreach channels. In this study, we estimated the financial and economic costs of the COVID-19 vaccination programme in the first year of roll out from February 2021 to January 2022 and one month prior, in one district of South Africa, the West Rand district.

**Methods:**

Financial and economic costs were estimated from a public payer’s perspective using top-down and ingredient-based costing approaches. Data were collected on costs incurred at the national level and from the West Rand district. Total cost and cost per COVID-19 vaccine dose were estimated for each of the five delivery channels implemented in the district. In addition, we estimated vaccine delivery costs which we defined as total cost exclusive of vaccine procurement costs.

**Results:**

Total financial and economic costs were estimated at US$8.5 million and US$12 million, respectively; with a corresponding cost per dose of US$15.31 (financial) and US$21.85 (economic). The two biggest total cost drivers were vaccine procurement which contributed 73% and 51% to total financial and economic costs respectively, and staff time which contributed 10% and 36% to total financial and economic costs, respectively. Total vaccine delivery costs were estimated at US$2.1 million (financial) and US$5.7 million (economic); and the corresponding cost per dose at US$3.84 (financial) and US$10.38 (economic). Vaccine delivery cost per dose (financial/economic) was estimated at US$2.93/12.84 and US$2.45/5.99 in hospitals and PHCs, respectively, and at US$7.34/20.29, US$3.96/11.89 and US$24.81/28.76 in fixed, temporary and mobile outreach sites, respectively. Staff time was the biggest economic cost driver for vaccine delivery in PHCs and hospitals while per diems and staff time were the biggest economic cost drivers for vaccine delivery in the three outreach delivery channels.

**Conclusion:**

This study offers insights for budgeting and planning of COVID-19 vaccine delivery in South Africa’s public healthcare system. It also provides input for cost-effectiveness analyses to guide future strategies for maximizing vaccination coverage in the country.

**Supplementary Information:**

The online version contains supplementary material available at 10.1186/s12913-024-11251-1.

## Background

South Africa started rolling out a national COVID-19 vaccination programme in February 2021 [1]. Given the devasting impact COVID-19 had on the country's population, the healthcare system and the economy [2], the national government committed approximately 15% of its total health budget between March 2021 and February 2022 to the COVID-19 vaccination programme [3]. However, due to global COVID-19 vaccine shortages, a risk-based phased approach was adopted [1]. First, healthcare workers were targeted through the Sisonke trial using the Johnson and Johnson’s (J&J) Janssen vaccine [1, 4]. From May 2021, the vaccination programme was rolled out to the general population, first targeting adults aged 60 years and older, other essential workers and subsequently, other age groups sequentially with both the Comirnaty (by Pfizer-BioNTech) and Johnson and Johnson (J&J) Janssen vaccines [4]. By December 2021, the target population had been extended to include all individuals aged 12 years and older [1].

The rollout of the COVID-19 vaccination programme was unprecedented in scale and especially in pace, given the urgency to achieve a 70% target vaccine coverage by December 2021 in the general population including sub-populations not traditionally targeted for vaccination [1, 3]. To maximise coverage and address vaccine equity for hard-to-reach rural populations, in the first year of the programme, COVID-19 vaccines were delivered to target populations via five differentiated delivery channels across public, private and non-governmental organisations (NGOs). These included hospitals, primary healthcare (PHC) facilities, fixed, temporary, and mobile outreach delivery channels.

Scale up of a national life-course vaccination programme relies on cost-effective and sustainable vaccine delivery models and the choice of COVID-19 delivery modality has implications not only for equitable vaccination coverage but also for the costs and cost-effectiveness of the vaccination programme. In addition, accurate budgeting and planning for the ongoing COVID-19 vaccine rollout to non-traditionally targeted subpopulations in South Africa require a thorough understanding of vaccination programme costs. There is, however, a dearth of evidence on real-world costs of COVID-19 vaccination programmes in low- and middle-income settings including South Africa. Many studies in these settings have adopted a normative approach, based on the application of guidelines and assumptions to describe and estimate, prospectively, vaccination programme costs ahead of real-world rollouts [5–7]. Crucially, some of these studies assumed the rollout of the programme on singular delivery platforms, thus potentially resulting in biased estimates of the cost of the COVID-19 vaccination programme.

In this study, we retrospectively estimated total financial and economic cost of the COVID-19 vaccination programme from a public payer’s perspective in the first year of programme rollout in one district of South Africa, the West Rand district. The district has a total population of approximately 770,000 and by January 2022, had achieved a COVID-19 vaccination coverage in eligible populations (individuals over the age of 12 years) of approximately 50% for those who had received at least 1 dose of either of the two COVID-19 vaccines and 41% for those fully vaccinated.

Financial costs captured the costs of resources that were paid for by the public payer which consisted of cash outlays incurred in the delivery of the COVID-19 vaccination programme [8, 9]. Economic costs, on the other hand, captured both financial costs and the opportunity costs of existing or donated resources [8, 9]. In addition, we estimated cost per vaccine dose of the five delivery channels to assess the most cost-efficient vaccine delivery channel. These estimates will not only be useful for informing ongoing discussions on the evolution of the COVID-19 vaccination programme in South Africa but also for informing budgeting and planning for the rollout of future large-scale vaccination programmes, such as potential tuberculosis, HIV and malaria vaccines targeted at wider population groups as well as for informing planning for pandemic preparedness.

### The COVID-19 vaccination programme in South Africa’s West Rand district

**Fig. 1:**
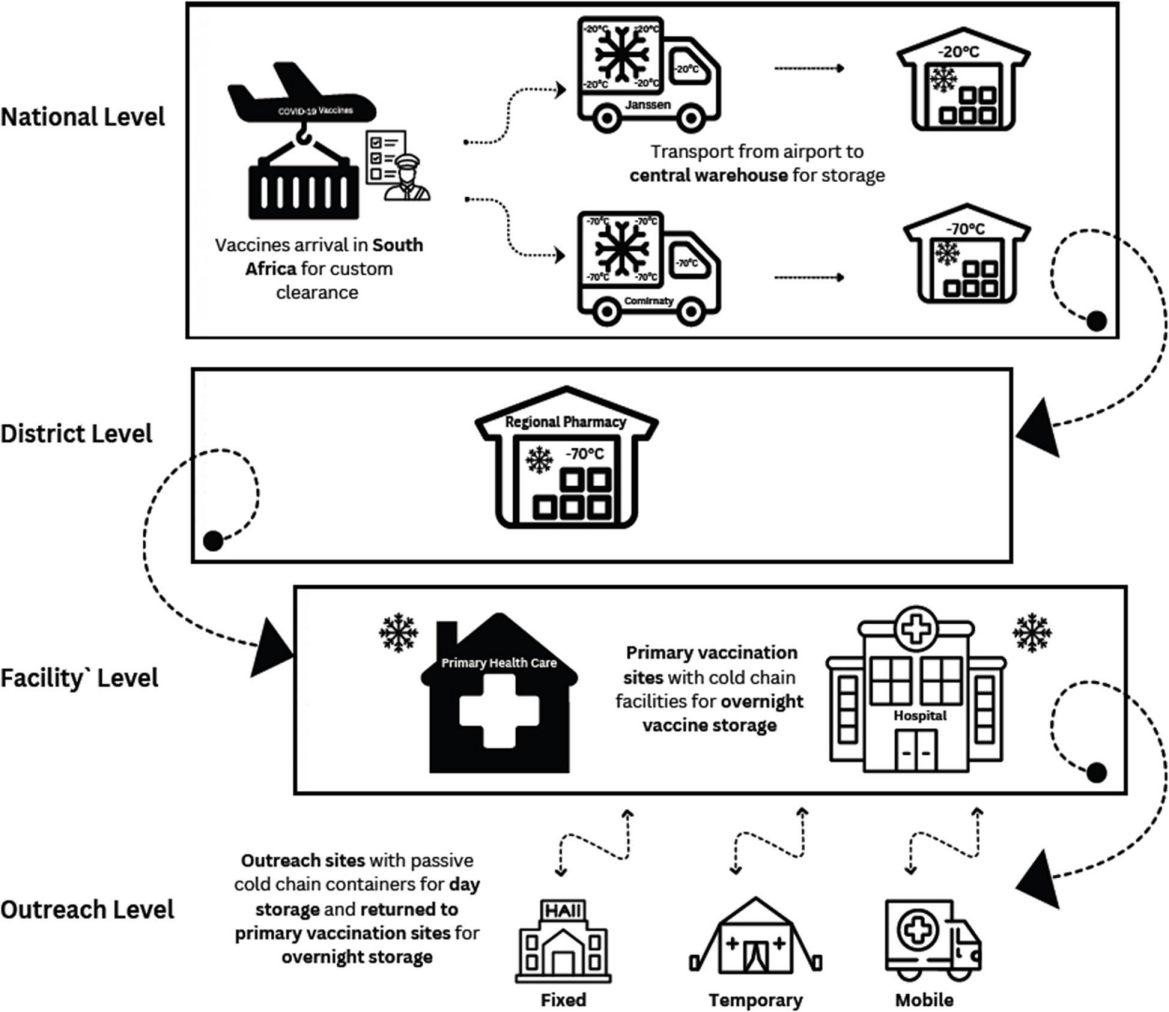
Distribution of COVID-19 vaccines in the West Rand district, January 2021 – January 2022

In the West Rand District, COVID-19 vaccines were administered via five delivery channels including existing health facilities—hospitals and PHC facilities, and three outreach channels—fixed, temporary, and mobile outreach channels. Fixed outreach sites were donated non-health facilities or mass vaccination venues such as sports centres, community town halls, churches, and school halls which provided only COVID-19 vaccination services. Temporary and mobile outreach channels offered vaccination services through roving and mobile teams, respectively. Temporary outreach channels provided COVID-19 vaccines on a temporary basis from locations which varied day-to-day based on identified need or priority groups and included sites such as old age homes, shopping malls and community town halls. Mobile outreach services were offered by a team of health personnel from within rented vans which served as mobile clinics. In addition to providing COVID-19 vaccination services, mobile outreach delivery channels were used to deliver other PHC services. Hospitals and PHC facilities were regarded as primary vaccination sites (Fig. 1) where COVID-19 vaccines were stored overnight and served as hubs to support the three outreach delivery channels, where vaccines required only for the day of service provision were stored in WHO-approved cooler boxes. While primary vaccination sites typically provided vaccination services within normal working hours, the three outreach channels provided vaccination services both within and outside normal work hours including on weekends and public holidays to increase the reach of the vaccination programme to eligible populations. As a result, health personnel in the outreach delivery channels received per diems for working overtime. Overall, COVID-19 vaccines were administered in the district in four hospitals, forty-seven PHC facilities, forty-two fixed outreach vaccination sites, four mobile clinics and by twelve roving temporary outreach teams.

Vaccine procurement was coordinated by the national government and distributed from central and regional warehouses to the West Rand district regional pharmacy, from where vaccines were distributed to COVID-19 vaccination sites (Fig. 1). Implementation of the COVID-19 vaccination programme in the district was coordinated by existing district health personnel through regular supervisory visits to vaccination sites, provision of in-service training to vaccination site personnel and overall monitoring, evaluation and collation of vaccination programme statistics using a de novo digital health information system, the Electronic Vaccination Data System (EVDS) specifically launched for the COVID-19 vaccination programme by the South African National Department of Health (NDoH). To facilitate district-level activities, additional resources such as rented vehicles, transportation, and overtime allowances (per diems) were provided to district health personnel. The district was also responsible for COVID-19 waste disposal which involved the use of rented vehicles for the collection and transportation of COVID-19 waste from all vaccination sites to a central point, from where they were disposed of by a contracted private company.

 COVID-19 vaccines were stored centrally within the district in the regional pharmacy using newly purchased cold chain equipment and supervised by district health pharmacists. At the district level, both the Comirnaty (by Pfizer-BioNTech) and J&J Janssen were stored under similar temperature conditions and both vaccines were distributed to vaccination sites using rented vehicles, also under similar temperature conditions. At the vaccination site level, additional cold chain equipment was purchased or obtained through donations to supplement existing cold chain equipment. These included cold chain accessories (such as digital fridge thermometers, ice packs and locks), active cold chain equipment (such as freezers and refrigerators) that required external sources of power supply and passive cold chain equipment (such as cooler boxes) that did not require external power supply. Existing generators were serviced and maintained to provide back-up power supply for active cold chain equipment within health facilities.

To minimize the impact of the COVID-19 vaccination programme on the provision of other healthcare services, additional human resources including temporary contract personnel and volunteers were mobilised, and deployed to provide vaccination services in outreach delivery channels. These personnel were supported and supervised by existing department of health-employed personnel at the district and health facility level. Overall, the number and composition of personnel varied between the five delivery channels with some personnel, such as district-level personnel, shared across the five delivery channels (Table A1).

## Methods

We estimated the financial and economic costs of COVID-19 vaccination programme from a public payer’s perspective using a combination of top-down and ingredient-based costing approaches.^1^ We estimated costs during the first year of the programme (February 2021-January 2022) and one month prior to the implementation of the programme to capture costs incurred in planning for programme rollout. All costs were collected in South African Rands (ZAR) and converted to 2021 US$ using an average exchange rate of US$1 to ZAR14.78.^2^

### Data collection

Costs incurred at three health system administrative levels (national-level, district-level and vaccine delivery channel-level) were collected from multiple sources using standardised questionnaires [10, 11], adapted to the South African setting. Data were collected retrospectively, by programme activities described in Table 1.

**Table 1:** Description of programme activities and resource inputs

Programme activity	Programme activity description	Resource inputs
Planning	All planning activities conducted one month prior to vaccine rollout (treated as capital resources) and ongoing programme management activities conducted from February 2021 to January 2022 (treated as recurrent resources)	• Staff time- time spent by different staff cadres at all administrative levels^a^. Time spent by staff at national level treated as capital cost • Vehicles and transport-vehicle rental costs and fuel incurred at district- and all vaccination channels except PHC sites • Per diem- overtime and transport costs incurred by district-level staff only
Vaccine procurement	Vaccine importation ( Comirnaty (by Pfizer-BioNTech) and J&J Janssen)	• Vaccine- cost of administered and wasted vaccine doses
Vaccine storage and distribution	Transportation from airport to central stores, warehousing in central stores, and distribution of COVID-19 vaccines from central stores to regional pharmacies; warehousing and distribution of COVID-19 vaccines from regional pharmacies to primary vaccination sites (hospitals and PHC); warehousing and distribution of vaccines to and from primary vaccination sites to outreach sites	• Staff time- time spent by different staff cadres at all administrative levels. Time spent by staff at national level treated as capital cost • Vehicles and transport-vehicle rental costs and fuel used for distribution of vaccines from airport to central /regional stores and to all vaccination sites • Per diem—overtime payment of district-level, mobile and fixed outreach staff • Cold chain equipment and supplies- incurred at district and vaccination sites such as WHO box cooler vaccine storage, ice packs, locks, digital fridge thermometers, minus 40-degree refrigerators, cold chain power supply
Cold chain maintenance	Maintenance of cold chain used by the COVID-19 vaccination programme; monitoring of cold chain equipment	• Staff time- time spent by different staff cadres at all administrative levels. Time spent by staff at national level treated as capital cost • Vehicles and transport-vehicle rental costs and fuel for supervisory visits by district level staff • Per diem—overtime payment for district-level staff
Advocacy, communication and social mobilisation	Social mobilisation and demand creation activities conducted within communities by district and vaccination site staff. Also includes coordination and monitoring by personnel at national level	• Staff time- time spent by different staff cadres at all administrative levels. Time spent by staff at national level treated as capital cost • Vehicles and transport-vehicle rental costs and fuel used by district level staff only • Per diem—per diem and overtime payment to staff of the three outreach sites and district level personnel, • Equipment and supplies- printing of IEC materials for social mobilisation • Other expenses communication expenses, advertisements, television commercials, billboard
Training	Formal training of staff via online Knowledge Hubs and in-service training conducted by district-level staff	• Staff time- time spent by different staff cadres at all administrative levels attending, delivering, or coordinating training events. Time spent by staff at national level treated as capital cost • Vehicles and transport-vehicle rental costs and fuel incurred by district-level staff only • Per diem overtime payment for district-level staff only • Equipment and supplies- printing cost for IEC training materials
Service delivery (vaccine administration)	All activities relating to the provision of vaccination services within vaccination sites	• Staff time- time spent by different staff cadres at delivery channel level only • Vehicles and transport-vehicle rental costs and fuel used for mobile, and temporary outreaches only • Per diem—per diem payments for outreach staff • Equipment and supplies- some examples are provided in Tables A2- 3 • Consumables- some examples are provided in Table A2 • Other expenses- rental value of fixed outreach venues
Supervision	General supervisory activities conducted by staff at all administrative levels	• Staff time- time spent by different staff cadres at all administrative levels. Time spent by staff at national level treated as capital cost • Vehicles and transport-vehicle rental costs and fuel used for general supervisory visits by district level staff • Per diem overtime payments for supervisors at district and vaccination site level
Waste management	Disposal of medical waste including COVID-19 vaccine vials, sharps and cotton wool	• Staff time- time spent by different staff cadres at all administrative levels. Time spent by staff at national level treated as capital cost • Vehicles and transport-vehicle rental costs and fuel used for transporting waste from sites to central location • Per diem—overtime payment to district level staff • Consumables- refuse bags, sharps disposal and medical waste box • Other expenses- collection and disposal of waste from central locations within the district
Vaccine safety surveillance and management of AEFI	Activities relating to planning, identification/detection, management of, notification/reporting of AEFI	• Staff time- time spent by different staff cadres at all administrative levels. Time spent by staff at national level treated as capital cost • Vehicles and transport-vehicle rental costs and fuel costs incurred by district level staff • Per diem overtime payment incurred by district-level staff
Record keeping; monitoring and evaluation	Activities related to the recording and monitoring of vaccine doses	• Staff time- time spent by different staff cadres at all administrative levels. Time spent by staff at national level treated as capital cost • Vehicles and transport-vehicle rental costs and fuel incurred by district-level staff only • Per diem- overtime payment costs for district-level staff • Equipment and supplies- Pocket Z-fold leaflet, vaccine card, stationary, printing materials, tablets, Wi-Fi routers and EVDS costs
Other campaign activities	Other campaign activities not captured in the other programme activities	• Staff time • Vehicles and transport • Per diem • Equipment and supplies • Other expenses

^a^Administrative levels include national, district, and delivery channels

*AEFI* Adverse event following immunisation

Data on costs incurred only at the national level (i.e. national-specific costs) were collected, from January 2021 to January 2022, through interviews with NDOH COVID-19 vaccination programme coordinators. The first doses of the COVID-19 vaccines in the district were administered in May 2021. Therefore, we collected district- and channel-level data from April 2021 to January 2022.

At national level, data collected included vaccine procurement costs, personnel time costs, EVDS cost and national-level vaccine transportation and storage costs. Vaccine procurement was done at the national level and the cost per dose of the Comirnaty (by Pfizer-BioNTech) and J&J Janssen vaccines were obtained from secondary sources and assumed to include air freight costs [1, 12]. For personnel time, data was collected on the proportion of time spent on each programme activity and on the total number of hours worked per day on the COVID-19 vaccination programme during our study period. Staff time by programme activity was then estimated by multiplying proportion of time spent on each programme activity by total hours worked, and valued using public sector salaries obtained from secondary sources [13]. For EVDS costs, a top-down approach was adopted. Total cost of implementing and maintaining the EVDS during our study period were obtained from the NDoH and allocated to initial capital investment and on-going human resource support using allocation factors (30% and 70%, respectively) provided by NDoH key informants.

At the district- and channel-levels, an ingredient-based costing approach was adopted. This involved the collection of data on quantities and unit costs (market prices) of resources used in the vaccination programme. District-and channel-level data was collected from one urban/semi-urban district, the West Rand district, located in the Gauteng province of South Africa.

Interviews were conducted with the district COVID-19 vaccination programme coordinator who in turn, consulted with other colleagues in collating data on quantities, unit costs and allocation of resources used, by programme activities (Tables A2 and 3). These included costs incurred only at the district level (district-specific costs) and costs incurred only at the delivery channel level (channel-specific costs). For channel-specific costs, data were collected on total quantity of resources used in all vaccination sites within each delivery channel. For example, during our study period there were four hospitals, forty-seven PHC facilities, and forty-two fixed outreach vaccination sites in the district, for which aggregate data was collected for each category of delivery channel. Similarly to data collection at the national level, personnel time was collected on the proportion of hours worked on each programme activity and the total number of hours worked on only the COVID-19 vaccination programme during our study period. In addition, data was collected on the total number of personnel receiving per diem and average per diem rate (Table A1). Unit costs of all consumables, equipment, supplies, and personnel salaries were obtained from COVID-19 vaccination programme expenditure records provided by the district programme coordinator. Finally, data were obtained from the EVDS database via the district programme coordinator on the total number of COVID-19 vaccine doses administered within each delivery channel and the corresponding number of doses wasted.

**Table 2:** Total costs (US$) and cost per dose (US$) by delivery channel^a^, West Rand district, January 2021-January 2022

	**Total doses administered**	**Total number of vaccination sites**	**Financial**			**Economic**		
			**Total costs**	**%**	**Cost per dose**	**Total costs**	**%**	**Cost per dose**
**Delivery Channel**^**a**^			**Excluding vaccine procurement cost (vaccine delivery cost)**					
Hospital	48 804	4	143 075	7%	2.93	626 512	11%	12.84
PHC facilities	326 415	47	800 317	38%	2.45	1 956 499	34%	5.99
Fixed outreach	116 271	42	853 552	40%	7.34	2 359 658	41%	20.29
Temporary outreach	56 752	12	224 831	11%	3.96	674 906	12%	11.89
Mobile outreach	3 864	4	95 882	5%	24.81	111 130	2%	28.76
West Rand District (total)	552 106		2 117 656	100%	3.84	5 728 704	100%	10.38
**Delivery Channel**			**Including vaccine procurement cost (total programme cost)**					
Hospital	48 804	4	696 896	8%	14.28	1 180 333	10%	24.19
PHC facilities	326 415	47	4 637 897	55%	14.21	5 794 079	48%	17.75
Fixed outreach	116 271	42	2 138 903	25%	18.40	3 645 009	30%	31.35
Temporary outreach	56 752	12	839 423	10%	14.79	1 289 498	11%	22.72
Mobile outreach	3 864	4	138 578	2%	35.86	153 826	1%	39.81
West Rand District (total)	552 106		8 451 699	100%	15.31	12 062 747	100%	21.85

^a^National and district level-specific cost have been allocated to each delivery channel

*PHC* Primary healthcare

**Table 3:** Total costs (2021 US$) and percent contribution to total costs by programme activity, West Rand district^a^, January 2021-January 2022

	**Financial**			**Economic**		
**Programme Activity**	**Total costs (US$)**	**%**	**Cost per dose (US$)**	**Total costs (US$)**	**%**	**Cost per dose (US$)**
Training	18 036	0.002%	0.03	130 393	1%	0,24
Planning	24 361	0.003%	0.04	174 110	1%	0,32
Other campaign activities	43 911	1%	0.08	214 509	2%	0,39
Waste management	74 355	1%	0.13	222 028	2%	0,40
Vaccine safety surveillance and AEFI management	37 787	0.004%	0.07	241 859	2%	0,44
Supervision	159 008	2%	0.29	392 024	3%	0,71
Cold chain maintenance	104 884	1%	0.19	407 003	3%	0,74
Advocacy, communication and social mobilisation	232 905	3%	0.42	606 893	5%	1,10
Vaccine storage and distribution	297 619	4%	0.54	711 820	6%	1,29
Record keeping, monitoring and evaluation	377 264	4%	0.68	1 158 490	10%	2,10
Service delivery (Vaccine administration)	878 357	10%	1.59	1 600 407	13%	2,90
Vaccine Procurement	6 203 212	73%	11.24	6 203 212	51%	11,24
Total	8 451 699	100%	15.31	12 062 747	100%	21.85

^a^Inclusive of national and district level-specific costs

*AEFI* Adverse event following immunization

### Data analysis and cost outcomes

We estimated total financial and economic costs of the COVID-19 vaccination programme, disaggregated by resource input categories and programme activities. In addition, we estimated total vaccine delivery costs which we defined as total costs exclusive of vaccine procurement costs. Total cost of each resource input was estimated by multiplying the unit cost of each resource by its respective quantity and resource allocation factor, where applicable. Under each programme activity, resource inputs were classified as capital/start-up resources (defined as inputs with a useful life greater than one year) and recurrent resources (defined as inputs with a useful life less than one year). Annual financial costs of capital resources were estimated using the straight-line depreciation approach [9]. Annual economic cost of capital resources was estimated by applying an annuity factor [9, 14] estimated using a discount rate of 5%, in line with NDoH methods guide [15, 16] and an assumed useful life for each capital resource obtained from existing literature [10]. For annualization of capital investment in the EVDS, we assumed a 3-year useful life, based on the assumption that the EVDS system would solely be used for the COVID-19 vaccination programme during this period, after which it would be repurposed to support the entire Extended Programme on Immunisation. Similarly, personnel time spent on planning for vaccine introduction one month prior to the rollout of the vaccination programme was assumed to be a capital investment and was annualized assuming a 3-year useful life. This was based on the assumption that the COVID-19 vaccination programme will likely taper off at the end of 3 years and the intensity of planning seen prior to the rollout of a new vaccine to population groups previously not targeted for vaccination, would not likely be repeated during this time. The impact of the assumed 3-year useful life on total cost was assessed in a sensitivity analysis as well as the impact of assuming planning costs to be capital –our sensitivity analysis is described below.

Resources shared by programme activities within the COVID-19 vaccination programme were allocated using allocation factors (Tables A2 and 3) collected during key informant interviews. Resources shared with non-COVID programmes were similarly allocated using allocation factors. For example, within our study district, mobile outreach channels provided both routine primary healthcare services and COVID-19 vaccines. Consequently, 50% of shared resources (vehicle cost, cold chain, and staff time) were allocated to the COVID-19 vaccination programme.

Total cost incurred at the study district was estimated as the sum of district-specific costs, channel-specific costs and a share (dose-weighted; Eq. 1) of national-specific costs. National-specific costs were apportioned to the study district using the district share of doses administered nationally within the public sector (Eq. 1). Total number of doses administered nationally via public delivery channels were obtained from the EVDS [17].

1 $$\text{District share of national-specific cost}={Total\;cost}_N\ast\frac{{Total\;doses}_D}{{Total\;doses}_N}\;$$

where *D* = district and* N* = national

Similarly, total cost of each delivery channel was estimated as the sum of channel-specific costs and a share (dose-weighted) of national- and district-specific costs. Finally cost per dose was estimated for the district as a whole and for each delivery channel (Eq. 2).

2 $${Cost\;per\;dose}_i=\frac{{Total\;cost}_i}{{Total\;doses}_i}$$

where, *i* = district, hospital, PHC, fixed, temporary and mobile delivery channels

### Sensitivity analysis

Using a one-way sensitivity analysis, we assessed the robustness of our findings to variations in discount rate and useful life assumed in the estimation of annual economic cost of the EVDS and personnel time spent on planning activities prior to programme implementation. Given the uncertainty in the anticipated lifetime of the COVID-19 vaccination programme in South Africa, in the basecase analysis we assumed a useful life of 3 years for capital investment in the EVDS and personnel time spent on planning activities one month prior to vaccine rollout. In the sensitivity analysis, we estimated total costs and cost per dose using alternative useful life of 1.5 years and 5 years while holding all other cost inputs constant. In addition, we assessed the impact of reducing the discount rate (from 5 to 3%) used in the estimation of annuity factors in line with recommendations from the International Decision Support Initiative (IDSI) [18]. Finally, we re-estimated total cost and cost per dose when personnel time spent on planning activities prior to vaccine introduction are treated as recurrent inputs.

## Ethics approval

Ethics approval for this study was obtained from the University of Witwatersrand Human Research Ethics Committee (Medical)- clearance certificate number: M210747.

## Results

### Total costs

Total financial and economic costs of the COVID-19 vaccination programme in our study district were estimated at approximately US$8.5 million and US$12 million, respectively (Table 2). This included delivery channel-specific costs, district-specific costs, and the district share of national-specific costs (Table 2). Total financial and economic vaccine delivery costs were estimated at US$2.1 million and US$5.7 million, respectively (Table 2), and were highest in fixed outreach (approximately US$853,550 and US$2.4 million, respectively, Table 2) and PHC delivery channels (approximately US$800,300 and US$1.96 million, respectively, Table 2).

Table 3 presents the distribution of total costs by programme activities for the entire district across all delivery channels. Overall, the biggest programmatic cost drivers were vaccine procurement, which accounted for 73% and 51% of total financial and economic costs, respectively; and vaccine service delivery which accounted for 10% and 13% of total financial and economic costs, respectively (Table 3).

The distribution of programme activity costs by administrative level (Table A4, Figure A1) shows that district-specific financial costs were largely driven by advocacy, communication, and social mobilisation costs (44%), while record keeping, monitoring and evaluation contributed the highest (31%) to total economic costs (Table A4).

**Table 4:** Total costs (2021 US$) and percent contribution to total costs by resource type, West Rand district^a^, January 2021-January 2022

	**Financial**			**Economic**		
**Resource type**	**Total costs (US$)**	**%**	**Cost per dose (US$)**	**Total costs (US$)**	**%**	**Cost per dose (US$)**
Other expenses	30 973	0.4%	0.06	82 007	1%	0.15
Vehicles and transport	191 971	2%	0.35	191 766	2%	0.35
Consumables	191 901	2%	0.35	191 901	2%	0.35
Equipment and supplies	210 874	2%	0.38	240 171	2%	0.44
Per diem	751 921	9%	1.36	751 921	6%	1.36
Staff time	870 848	10%	1.58	4 401 769	36%	7.97
Vaccine (plus wastage)	6 203 212	73%	11.24	6 203 212	51%	11.24
Total	8 451 699	100%	15.31	12 062 747	100%	21.85

^a^Inclusive of national and district level-specific costs

In PHC and hospital delivery channels, vaccine administration, as well as record keeping, monitoring and evaluation contributed the highest to total financial and economic costs while in all three outreach channels, vaccine administration contributed the highest to total financial and economic costs (Table A4, Figure A1).

Table 4 presents distribution of cost by resource categories for the entire district across all delivery channels. Overall, vaccine cost was the biggest cost driver (Table 4). Staff time made the second biggest contribution to total cost, accounting for 10% and 36% of total financial and economic costs, respectively (Table 4). The distribution of resource costs by administrative levels showed that staff time contributed the highest to district-specific economic cost (78%, Table A5, Figure A2) while vehicles and transportation costs contributed the highest to district-specific financial cost (41%; Table A5, Figure A2). In hospital delivery channels, staff time was the biggest resource cost driver accounting for 62% and 91% of total financial and economic cost, respectively (Table A5). Similarly, in PHC delivery channels, staff time was the biggest cost driver accounting for 71% and 87% of total financial and economic costs, respectively (Table A5). In all three outreach delivery channels – per diem costs contributed the highest to total financial cost (Table A5). Total economic costs in fixed and temporary outreach delivery channels were largely driven by staff time (71% and 65% respectively; Table A5) while per diems contributed the highest to total economic costs in mobile outreach channels (54%; Table A5).

### Cost per dose

In our study district, a total of 552,106 doses were administered (Table 2), contributing 2% to the total number of doses administered nationally via public delivery channels (Table A6). Cost per dose administered in the district was estimated at US$15.31 (financial costs) and US$21.85 (economic costs; Table 2). This included the district share of national-specific costs, district-specific costs, and channel-specific costs. Vaccine delivery cost per dose was estimated at approximately US$3.84 (financial costs) and US$10.38 (economic costs; Table 2).

Across delivery channels, the highest number of doses were administered in PHCs (326,415; Table 2) and fixed outreach sites (116,271; Table 2), each contributing 59% and 21%, respectively, to total doses administered within the district (Table A6). In all five delivery channels, financial and economic costs, respectively, ranged from US$14.21 and US$17.75 per dose in PHC facilities to US$35.86 and US$39.81 per dose in mobile outreach sites (Table 2). This included each channel share of national- and district-specific costs as well as channel-specific costs. When vaccine procurement costs were excluded, vaccine delivery cost per dose (financial and economic cost, respectively) ranged from US$2.45 and US$5.99 in PHC facilities to US$24.81 and US$28.76 in mobile outreach channels (Table 2).

### Sensitivity analysis

Our results were robust to variations in discount rates assumed in estimating annual economic cost of all capital resources and uncertainty in useful life assumed in the estimation of EVDS and planning personnel annual economic costs. Decreasing discount rate from the basecase value of 5% to 3% marginally increased total cost and cost per dose administered (Figure A3). Similarly, varying useful life from 1.5 years to 5 years had minimal impact on total cost and cost per dose (Figure A4). The minimal impact of variations in discount rate and useful life on costs reflects the low contribution of capital costs to total cost which was largely driven by recurrent costs (Table A7). When planning activity costs were treated as recurrent resources, financial cost per dose increased marginally from US$15.31 to US$15.48 while economic cost per dose increased from US$21.85 to US$22.91 (Figure A5).

## Discussion

The COVID-19 pandemic required rapid scale up of the COVID-19 vaccination programme to protect populations from COVID-19 associated morbidity and mortality. As a result, COVID-19 vaccines were distributed via five delivery channels – hospitals, PHC, fixed outreach, mobile outreach, and temporary outreach delivery channels. This study assessed the financial and economic costs associated with implementing the COVID-19 vaccination programme in the West Rand district of South Africa via public health sector delivery channels. Overall, financial and economic vaccine delivery cost per dose in the district were estimated at US$3.84 and US$10.38, respectively. Variations were observed in cost per dose across the five delivery channels assessed with the highest vaccine delivery cost per dose estimated for mobile outreach vaccination sites (US$24.81 and US$28.76, financial and economic cost, respectively) and the lowest for PHC facilities (US$2.45 and US$5.99, financial and economic cost, respectively).

Consistent with findings from other settings [5, 7, 19–22], vaccine procurement was the biggest driver of total programme costs, while staff time was the biggest driver of vaccine delivery costs. Although additional temporary contract staff had been recruited to minimise the impact of the vaccination programme on existing resources, the high economic staff cost observed here demonstrates the reliance on existing personnel to deliver the vaccination programme and the potential implications this may have had on the delivery of other essential health services particularly in hospitals and PHC facilities. Per diems also made one of the biggest contributions to total cost and cost per dose administered. This was largely driven by per diems paid to outreach contract staff providing vaccination services out of normal working hours.

Overall, total costs were highest in PHC and fixed outreach channels with both delivery channels accounting for 80% of total doses administered in the district. However, cost per dose estimates suggests that vaccine rollout was more efficient via PHC channels compared to fixed outreach channels. Despite incurring comparable total costs, economic cost per dose administered in PHC facilities was 46% less than that of fixed outreach channels due to substantially fewer number of doses administered via fixed outreach sites. Overall, across all five delivery channels, the most efficient delivery channel was PHC channels while mobile outreach channels were the least efficient with an estimated vaccine delivery cost per dose that was substantially higher than that of PHC channels.

This study contributes to the growing number of published studies retrospectively estimating the costs of a COVID-19 vaccination programme in different context [20–22]. Prior to the global rollout of COVID-19 vaccines, normative approaches were adopted to prospectively estimate the costs of the COVID-19 vaccination programme in different context based on assumptions around resource quantities and unit costs [5, 7, 19]. For example, Liu et al. [5] estimated a cost per dose of US$16.13 for administering a Pfizer-like vaccine in South Africa through facility-based delivery channels. While this estimate is within the range of our estimated cost per dose (economic) for PHC (US$17.75) and hospital (US$24.19) channels, there are some divergences in unit cost estimates and assumptions made on the types and quantities of resources deployed. For example, Lui et al. [5] assumed a cost per vaccine dose of US$13.09 while we used a lower cost per dose of US$10.96 for the Pfizer vaccine based on published reports from the South Africa NDoH [1]. Furthermore, Liu et al. [5] assumed no increase in the workforce, while in practice, the workforce had been expanded to minimise the impact of the COVID-19 vaccination programme on the provision of other essential health services. As a result, in our study, staff time contributed the highest to both financial and economic costs in PHC and hospital delivery channels. Our study provides real-world estimates of a COVID-19 vaccination programme implemented at scale to rapidly reach targeted populations.

Our study has some limitations. First, our cost estimates were based on data collected from one district in South Africa which contributed 2% to total number of doses administered nationally via public delivery channels. As a result, our cost estimates are not representative of cost per dose across the entire country. Variations in the choices of resource inputs used across districts and demographic characteristics of districts may affect the type of delivery modality deployed as well as the number of doses administered via each channel, and consequently, cost per dose administered. However, given that our study district was based in the highest populated province in South Africa, there are wider implications of this study to other urban/semi-urban settings in the country.

Second, the allocation of shared resources across COVID-19 and non-COVID-19 vaccination programme activities may have been subject to recall bias given that data, including resources used at channel level, were collected retrospectively through interviews with district level staff.

Third, given that data collection was conducted at the district level, it is possible that reported quantities of some resources used within delivery channels, such as consumables, were total quantities delivered to each delivery channel and not the actual quantity of resources utilised. Conversely, we may not have captured quantities of pre-existing stockpiles of resources utilised within delivery channels. As a result, the quantities of some resources utilised at the channel levels may have been over- or under- stated.

Fourth, assumptions made about the useful life of programme-specific capital resources such as the EVDS, may have biased our estimates of programme costs. Although we assessed the impact of this in a sensitivity analysis, we cannot rule out future redeployment of these resources to other non-COVID 19 vaccination programmes within the useful life assumed in our study.

Fifth, our cost estimate represents the cost of delivering COVID-19 vaccines via public sector channels and does not capture the full costs of the vaccination programme in South Africa where, in addition to public sector delivery channels, 26% of total doses administered nationally within our study period were administered via private and NGO-run delivery sites. Nevertheless, our cost estimates are informative to public sector programme planners on the cost implications of the COVID-19 vaccination programme or of similar future vaccination programmes.

Finally, the study was conducted during the first year of the COVID-19 vaccination programme when the intensity of the programme was at its peak, both in terms of the deployment of resources and vaccine uptake [23]. Therefore, total cost and cost per dose reported here are unlikely to be a true reflection of cost estimates under non-pandemic conditions. For example, by the second year of roll out (from February 2022), the COVID-19 vaccination programme was gradually integrated into existing routine healthcare services offered via PHC facilities, hospitals and mobile clinics with fixed and temporary outreach services gradually rolled back. In addition, uptake of the COVID-19 vaccine had begun to wane [23]. Therefore, future studies may be needed to better understand the costs of the COVID-19 vaccination programme when delivered as part of routine services.

## Conclusion

This study affords insights into the costs and cost drivers of the COVID-19 vaccination programme in South Africa to inform ongoing budgeting and planning for COVID-19 vaccine delivery in the public healthcare system, particularly as the programme transitions to an integrated model. The findings also contribute valuable information for cost-effectiveness analyses, guiding future optimal delivery strategies for maximizing vaccination coverage across diverse population groups. However, additional estimates are needed as the programme fully integrates into the health system to understand the financial and economic cost implications of a routine life course vaccination programme.

### Supplementary Information

Supplementary Material 1.

## Data Availability

The datasets used and/or analysed during the current study are available from the corresponding author on reasonable request.

## Abbreviations

EVDS: Electronic Vaccination Data System
IDSI: International Decision Support Initiative
J&J: Johnson and Johnson
NDoH: National Department of Health
NGOs: Non-governmental organisations
PEPFAR: President’s Emergency Plan for AIDS Relief
PHC: Primary healthcare
USAID: United States Agency for International Development

## Footnotes

1 A CHEERS Checklist can be found in Supplemental Table A8.

2 https://www.resbank.co.za/en/home/publications/monthly-releases?category=Monthly%20Release%20of%20Selected%20Data&rows=25&year=2021&page=1
