# Supplementary material for: Costs of the COVID-19 vaccination programme: estimates from the West Rand district of South Africa, 2021/2022
Source: BMC Health Serv Res. 2024 Jul 29;24:857. doi: 10.1186/s12913-024-11251-1 (PMC11285413; doi:10.1186/s12913-024-11251-1)
Supplement: Supplementary file 1 — Supplementary Material 1. [file 12913_2024_11251_MOESM1_ESM.docx]

**Table A1- Categories of COVID-19 vaccination programme staff by delivery channels, West Rand district, January 2021 – January 2022**

| **Saff cadre** | **Hospital** | **PHC facilities** | **Fixed outreach** | **Temporary outreach** | **Mobile outreach** | **Average per diem rate (US$)** |  |  |  |  |
| --- | --- | --- | --- | --- | --- | --- | --- | --- | --- | --- |
| Vaccination site manager | 4 | - | 18 | - | - | - |  |  |  |  |
| Vaccine controller (Professional nurses) | - | 44 | 18 | - | - | - |  |  |  |  |
| Vaccine controller (Pharmacist) | 4 | 4* | | | |  |  |  | - |  |
| Vaccine controller  (District pharmacist) | 1* | | | | |  |  |  |  | - |
| Vaccine controller  (Pharmacy assistant) | 4 | 18 | - | - | - | - |  |  |  |  |
| Data capturer | 4 | 88 | 26 | 12 | 5 | - |  |  |  |  |
| COVID-19 Screening officer | - | 44 | 26 | - | - | - |  |  |  |  |
| Vaccinator  (Professional nurses) | 10 | 44 | 37 | 12 | 5 | 482.23 |  |  |  |  |
| Health promoter | - | 44 | 5 | 5 | 5 | 265.28 |  |  |  |  |
| Site/queue marshal | 16 | 29 | 18 | - | - | - |  |  |  |  |
| Vaccinator (Doctor) | 4 | 4 | 4 | 4 | 4 | 482.23 |  |  |  |  |
| Driver | 4 | 4 | 5 | - | 5 | 251.63 |  |  |  |  |
| Environmental health officer | 4 | 4 | 4 | 1 | 1 | - |  |  |  |  |
| EVDS scheduler | 2* | | | | | - |  |  |  | - |
| *Shared across delivery channels | | | | | |  |  |  |  |  |

**Table A2: Consumables and supplies quantity, allocation and unit cost**, **West Rand district, January 2021 – January 2022**

|  | **Hospitals** | | **PHC facilities** | | **Fixed Outreach** | | **Temporary Outreach** | | **Mobile Outreach** | | **Unit costs (US$)** |
| --- | --- | --- | --- | --- | --- | --- | --- | --- | --- | --- | --- |
|  | **Quantity** | **Allocation factor** | **Quantity** | **Allocation factor** | **Quantity** | **Allocation factor** | **Quantity** | **Allocation factor** | **Quantity** | **Allocation factor** |  |
| Pfizer (Comirnaty) doses administered | 47769 | 100% | 271029 | 100% | 99821 | 100% | 31901 | 100% | 2816 | 100% | 10.96 |
| J&J (Janssen) doses administered | 1035 | 100% | 55386 | 100% | 16450 | 100% | 24851 | 100% | 1048 | 100% | 10.00 |
| Vaccine card (prior to EVDS-issued vaccination certificates) | 10000 | 100% | 15000 | 100% | 15000 | 100% | 5000 | 100% | 5000 | 100% | 1.69 |
| Pocket Z-fold leaflet | 5000 | 100% | 7500 | 100% | 7500 | 100% | 2500 | 100% | 2500 | 100% | 1.42 |
| 21G needle | 49700 | 100% | 120000 | 100% | 100000 | 100% | 50000 | 100% | 3000 | 100% | 0.02 |
| 22G needle | - | - | 180000 | 100% | 20000 | 100% | 10000 | 100% | 1000 | 100% | 0.02 |
| 23G needle | - | - | 60000 | 100% | - | - | - | - | - | - | 0.01 |
| Sodium chloride 0.9% 2ml | 48600 | 100% | 300000 | 100% | 100000 | 100% | 32000 | 100% | 2900 | 100% | 0.20 |
| Water for injection | - | - | 25000 | 100% | - | - | - | - | - | - | 0.09 |
| Alcohol spray | 8 | 100% | 58 | 100% | 28 | 100% | 15 | 100% | 8 | 100% | 4.54 |
| Cotton wool packets | 600 | 100% | 8000 | 100% | 600 | 100% | 800 | 100% | 105 | 100% | 3.26 |
| Hydrogen peroxide | - | 100% | 2 | 100% | - | 100% | - | 100% | - | 100% | 0.60 |
| Sharps Container 20L | 318 | 100% | 2176 | 100% | 600 | 100% | 280 | 100% | 18 | 100% | 3.72 |
| Waste Box with red liner | 40 | 100% | 120 | 100% | 182 | 100% | 105 | 100% | 8 | 100% | 10.49 |
| Refuse bags black (packet of 50) | 50 | 100% | 1000 | 100% | 500 | 100% | 100 | 100% | 50 | 100% | 3.25 |
| Vehicles (Rented) | 4 | 50% | - | - | 4 | 50% | 10 | 47% | 4 | 15% |  |

**Table A3: Capital equipment resource quantity. allocation and unit cost**

|  | **Hospitals** | | **PHC facilities** | | **Fixed Outreach** | | **Temporary Outreach** | | **Mobile Outreach** | | **Unit costs (US$)** | **Useful life years** |
| --- | --- | --- | --- | --- | --- | --- | --- | --- | --- | --- | --- | --- |
|  | **Quantity** | **Allocation factor** | **Quantity** | **Allocation factor** | **Quantity** | **Allocation factor** | **Quantity** | **Allocation factor** | **Quantity** | **Allocation factor** |  |  |
| MTN MiFI Rounter (Model: MZ423) | - | - | - | - | 5 | 70% | 5 | 70% | - | - | 33.76 | 2 |
| Samsung Galaxy Tab A | 15 | 70% | - | - | - | - | - | - | - | - | 202.90 | 2 |
| Huawei MediaPad T3 7 | - | - | - | - | 10 | 70% | 6 | 70% | - | - | 202.90 | 2 |
| Samsung Galaxy Tab A (2016) | - | - | - | - | 1 | 70% | - | - | - | - | 202.90 | 2 |
| Lenovo Tab M10 | - | - | 50 | 70% | 4 | 70% | - | - | 4 | 70% | 169.14 | 5 |
| Huawei MediaPad M5 Lite | - | - | - | - | 20 | 70% | 23 | 70% | - | - | 439.69 | 2 |
| Huawei MatePad | 12 | 70% | 54 | 70% | - | - | - | - | - | - | 473.52 | 2 |
| Samsung Tab A7 | - | - | - | - | - | - | 4 | 70% | - | - | 202.90 | 2 |
| Compression planks | - | - | - | - | 24 | 100% | 5 | 100% | 5 | 100% | 49.75 | 5 |
| Jump bags (CPR) | - | - | - | - | 24 | 100% | 16 | 100% | - | - | 42.28 | 5 |
| Signages | 10 | 100% | 50 | 100% | 30 | 100% | 5 | 100% | 5 | 100% | 66.30 | 10 |
| Portable powered speakers with wireless microphone | - | - | - | - | 5 | 100% | 5 | 100% | - | - | 33.76 | 2 |
| Folding table | - | - | - | - | 10 | 100% | 40 | 100% | - | - | 21.11 | 10 |
| Chair | - | - | - | - | 30 | 100% | 120 | 100% | - | - | 9.47 | 5 |
| Steel Gazebo | - | - | 5 | 100% | - | - | 13 | 100% | 2 | 100% | 169.14 | 5 |
| Campmaster Gazebo | 2 | 70% | - | - | 4 | 70% | 10 | 70% | 4 | 70% | 246.06 | 5 |
| Tents | 2 | 100% | 5 | 100% | - | - | 13 | 100% | - | - | 981.00 | 5 |
| WHO Box cooler vaccine storage (newly purchased/donated) | 4 | 100% | 16 | 100% | 15 | 100% | 11 | 100% | 4 | 100% | 259.80 | 5 |
| WHO Box cooler vaccine storage (previously owned) | - | - | 80 | 50% | - | - | - | - | - | - | 259.80 | 5 |
| Minus 40 refrigerators (newly purchased/donated) | - | - | 8 | 80% | - | - | - | - | - | - | 4 758.39 | 10 |
| Minus 40 refrigerators (previously owned) | 9 | 45% | 88 | 48% | - |  | - | - | 4 | 100% | 4 758.39 | 10 |
| Freezer (-70) (newly purchased/donated) | 2 shared across the district at 100% usage for the COVID-19 vaccination programme | | | | | | | | | | 5 034.09 | 10 |
| Digital Fridge Thermometers (newly purchased/donated) | 20 | 100% | 200 | 100% | 50 | 100% | 60 | 100% | 20 | 100% | 84.57 | 10 |
| Locks (newly purchased/donated) | - | - | 25 | 100% | - | - | - | - | - | - | 4.06 | 5 |
| Ice packs (newly purchased/donated) | 200 | 100% | 800 | 100% | 500 | 100% | 400 | 100% | 50 | 100% | 0.45 | 2 |

# **Table A4-****Total cost (2021 US$) by administrative level and programme activity**

| **National-specific** |  | | | |
| --- | --- | --- | --- | --- |
|  | **Financial cost (US$)** | **Financial cost (%)** | **Economic Cost (US$)** | **Economic Cost (%)** |
| Vaccine procurement | 6 203 212 | 98% | 6 203 212 | 96% |
| Advocacy, communication, and social mobilisation | 0 | 0% | 107 | 0% |
| Cold chain maintenance | 0 | 0% | 12 | 0% |
| Planning | 0 | 0% | 752 | 0% |
| Record keeping, monitoring and evaluation | 13 648 | 0% | 110 801 | 2% |
| Supervision | 0 | 0% | 208 | 0% |
| Training | 0 | 0% | 136 | 0% |
| Vaccine safety surveillance and AEFI management | 0 | 0% | 125 | 0% |
| Vaccine storage and distribution | 130 832 | 2% | 130 937 | 2% |
| Waste management | 0 | 0% | 80 | 0% |
| Other campaign activities | 0 | 0% | 0 | 0% |
| **Total** | **6 347 692** | **100%** | **6 446 369** | **100%** |
| **District-specific** |  | | | |
|  | **Financial cost (US$)** | **Financial cost (%)** | **Economic Cost (US$)** | **Economic Cost (%)** |
| Advocacy, communication, and social mobilisation | 28 660 | 44% | 51 723 | 17% |
| Cold chain maintenance | 1 961 | 3% | 15 094 | 5% |
| Planning | 7 484 | 11% | 54 069 | 18% |
| Record keeping, monitoring and evaluation | 2 483 | 4% | 94 058 | 31% |
| Supervision | 6 383 | 10% | 34 749 | 11% |
| Training | 2 590 | 4% | 7 886 | 3% |
| Vaccine safety surveillance and AEFI management | 1 926 | 3% | 11 703 | 4% |
| Vaccine storage and distribution | 8 561 | 13% | 21 247 | 7% |
| Waste management | 5 359 | 8% | 10 259 | 3% |
| Other campaign activities | 0 | 0% | 1 576 | 1% |
| **Total** | **65 406** | **100%** | **302 364** | **100%** |
| **Hospital-specific** |  | | | |
|  | **Financial cost (US$)** | **Financial cost (%)** | **Economic Cost (US$)** | **Economic Cost (%)** |
| Advocacy, communication, and social mobilisation | 12 744 | 9% | 62 761 | 11% |
| Cold chain maintenance | 9 945 | 7% | 46 331 | 8% |
| Planning | 2 294 | 2% | 19 401 | 3% |
| Record keeping, monitoring and evaluation | 43 497 | 32% | 123 694 | 21% |
| Service delivery (Vaccine administration) | 45 107 | 33% | 146 518 | 25% |
| Supervision | 0 | 0% | 28 947 | 5% |
| Training | 460 | 0% | 9 019 | 2% |
| Vaccine safety surveillance and AEFI management | 9 945 | 7% | 25 645 | 4% |
| Vaccine storage and distribution | 5 957 | 4% | 75 035 | 13% |
| Waste management | 4 178 | 3% | 38 336 | 6% |
| Other campaign activities | 1 960 | 1% | 14 167 | 2% |
| **Total** | **136 087** | **100%** | **589 855** | **100%** |
| **Primary Healthcare-specific** |  | | | |
|  | **Financial cost (US$)** | **Financial cost (%)** | **Economic Cost (US$)** | **Economic Cost (%)** |
| Advocacy, communication, and social mobilisation | 50 571 | 7% | 181 466 | 11% |
| Cold chain maintenance | 76 033 | 10% | 157 956 | 9% |
| Planning | 8 094 | 1% | 32 326 | 2% |
| Record keeping, monitoring and evaluation | 216 728 | 29% | 452 143 | 26% |
| Service delivery (Vaccine administration) | 183 271 | 24% | 437 375 | 26% |
| Supervision | 17 861 | 2% | 31 373 | 2% |
| Training | 11 867 | 2% | 36 843 | 2% |
| Vaccine safety surveillance and AEFI management | 18 965 | 3% | 104 612 | 6% |
| Vaccine storage and distribution | 126 304 | 17% | 208 168 | 12% |
| Waste management | 43 722 | 6% | 68 902 | 4% |
| Other campaign activities | 162 | 0% | 162 | 0% |
| **Total** | **753 578** | **100%** | **1 711 327** | **100%** |
| **Fixed Outreach-specific** |  | | | |
|  | **Financial cost (US$)** | **Financial cost (%)** | **Economic Cost (US$)** | **Economic Cost (%)** |
| Advocacy, communication, and social mobilisation | 104 128 | 12% | 205 429 | 9% |
| Cold chain maintenance | 15 222 | 2% | 163 599 | 7% |
| Planning | 5 184 | 1% | 55 199 | 2% |
| Record keeping, monitoring and evaluation | 82 055 | 10% | 309 387 | 14% |
| Service delivery (Vaccine administration) | 430 663 | 51% | 713 482 | 31% |
| Supervision | 131 880 | 16% | 258 504 | 11% |
| Training | 1 341 | 0% | 47 647 | 2% |
| Vaccine safety surveillance and AEFI management | 4 526 | 1% | 62 155 | 3% |
| Vaccine storage and distribution | 16 089 | 2% | 236 759 | 10% |
| Waste management | 12 982 | 2% | 69 568 | 3% |
| Other campaign activities | 32 834 | 4% | 150 599 | 7% |
| **Total** | **836 903** | **100%** | **2 272 326** | **100%** |
| **Temporary Outreach-specific** |  | | | |
|  | **Financial cost (US$)** | **Financial cost (%)** | **Economic Cost (US$)** | **Economic Cost (%)** |
| Advocacy, communication, and social mobilisation | 25 754 | 12% | 93 232 | 15% |
| Cold chain maintenance | 0 | 0% | 21 163 | 3% |
| Planning | 643 | 0% | 10 980 | 2% |
| Record keeping, monitoring and evaluation | 4 843 | 2% | 53 262 | 8% |
| Service delivery (Vaccine administration) | 170 326 | 79% | 253 277 | 40% |
| Supervision | 1 163 | 1% | 35 396 | 6% |
| Training | 56 | 0% | 26 014 | 4% |
| Vaccine safety surveillance and AEFI management | 702 | 0% | 34 771 | 5% |
| Vaccine storage and distribution | 6 614 | 3% | 34 012 | 5% |
| Waste management | 6 112 | 3% | 31 756 | 5% |
| Other campaign activities | 493 | 0% | 38 416 | 6% |
| **Total** | **216 705** | **100%** | **632 279** | **100%** |
| **Mobile Outreach-specific** |  | | | |
|  | **Financial cost (US$)** | **Financial cost (%)** | **Economic Cost (US$)** | **Economic Cost (%)** |
| Advocacy, communication, and social mobilisation | 11 048 | 12% | 12 174 | 11% |
| Cold chain maintenance | 1 722 | 2% | 2 848 | 3% |
| Planning | 662 | 1% | 1 384 | 1% |
| Record keeping, monitoring and evaluation | 14 010 | 15% | 15 145 | 14% |
| Service delivery (Vaccine administration) | 48 990 | 51% | 49 754 | 46% |
| Supervision | 1 722 | 2% | 2 848 | 3% |
| Training | 1 722 | 2% | 2 848 | 3% |
| Vaccine safety surveillance and AEFI management | 1 722 | 2% | 2 848 | 3% |
| Vaccine storage and distribution | 3 263 | 3% | 5 663 | 5% |
| Waste management | 2 002 | 2% | 3 127 | 3% |
| Other campaign activities | 8 463 | 9% | 9 588 | 9% |
| **Total** | **95 328** | **100%** | **108 228** | **100%** |

**Figure A1- Cost per dose (2021 US$) by administrative level and programme activity**

*National level*

**
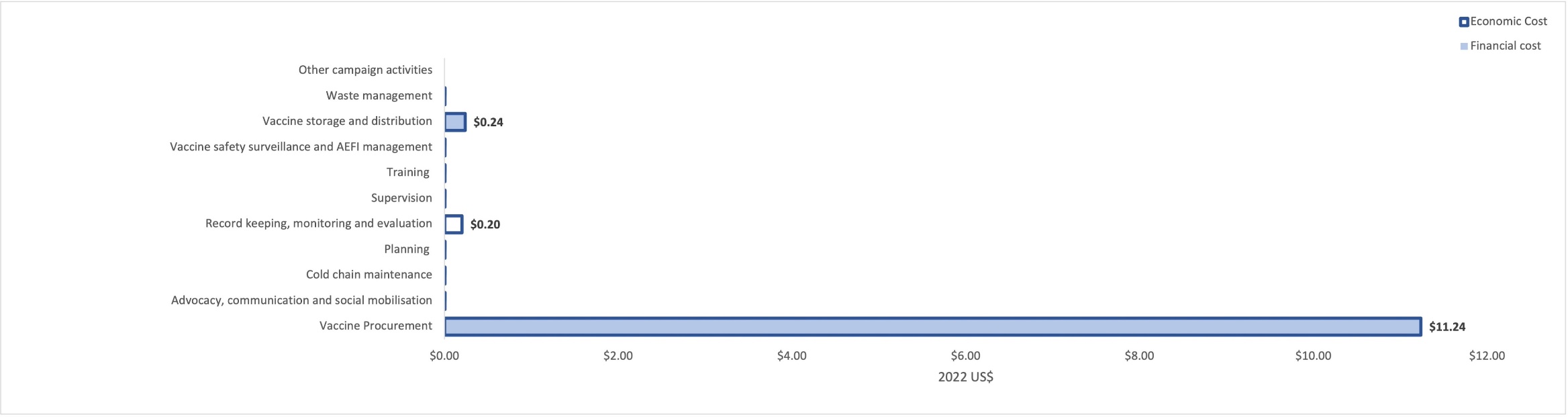
**

*District level*


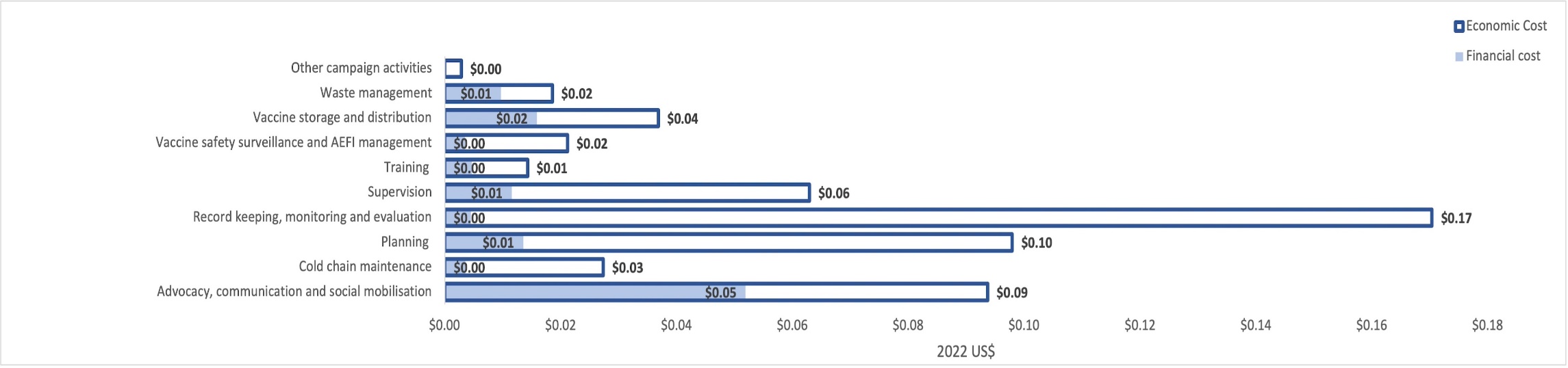


*Hospital level*


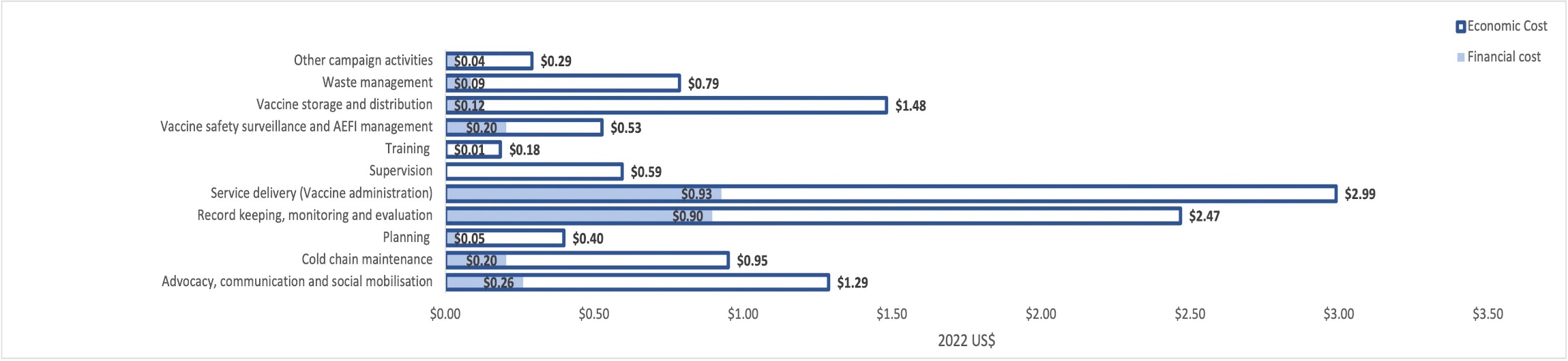


*Primary healthcare level*


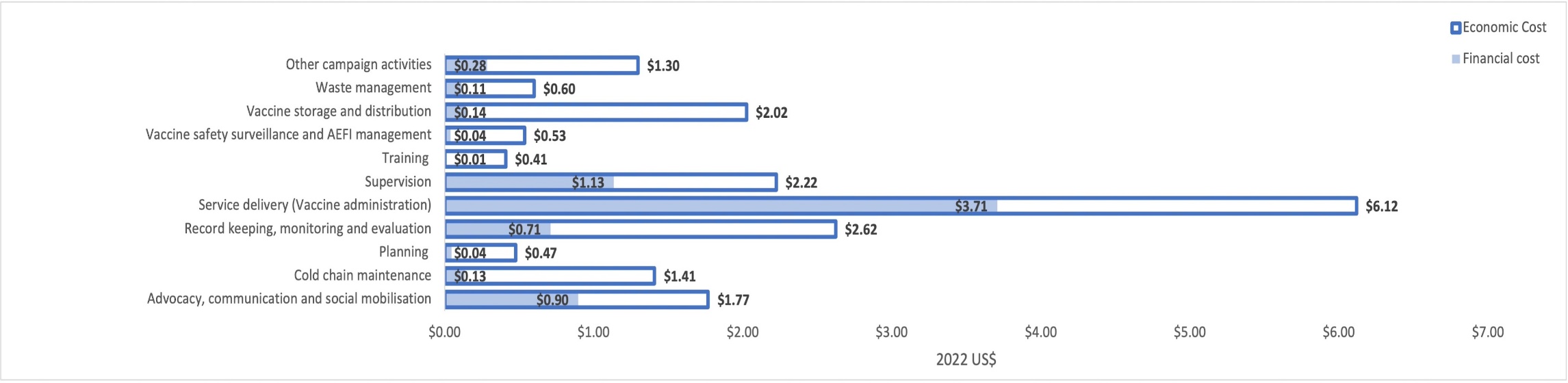


*Fixed outreach level*


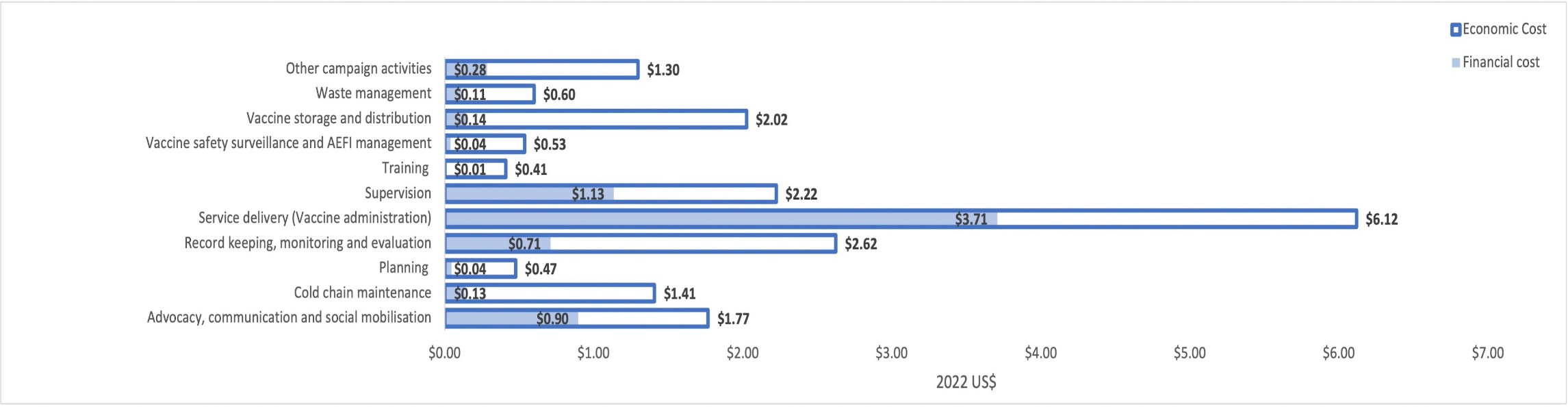


*Temporary outreach level*


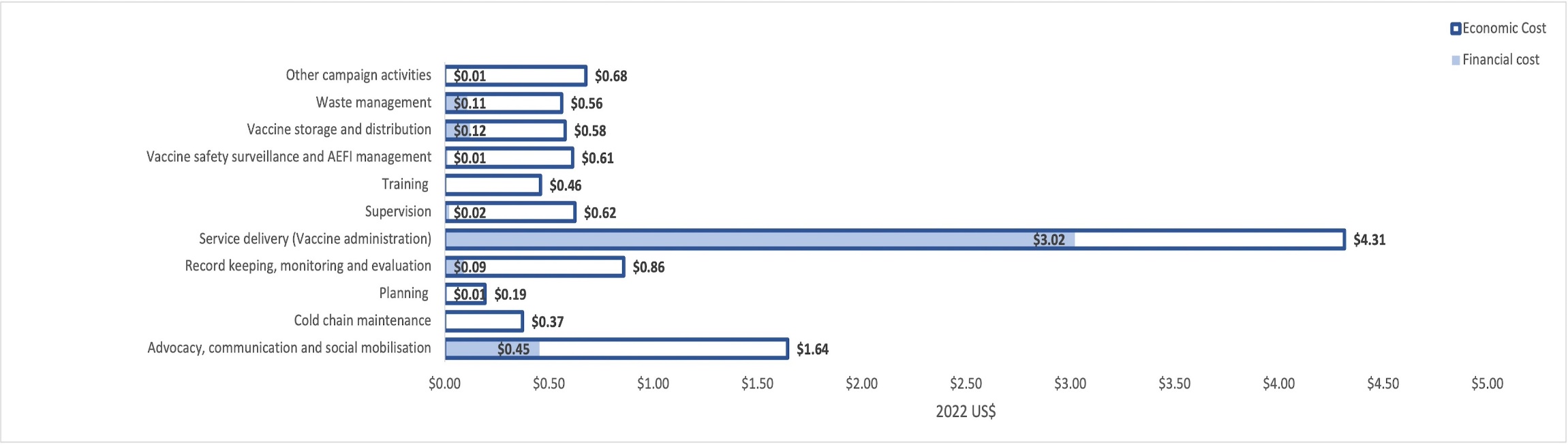


*Mobile outreach level*


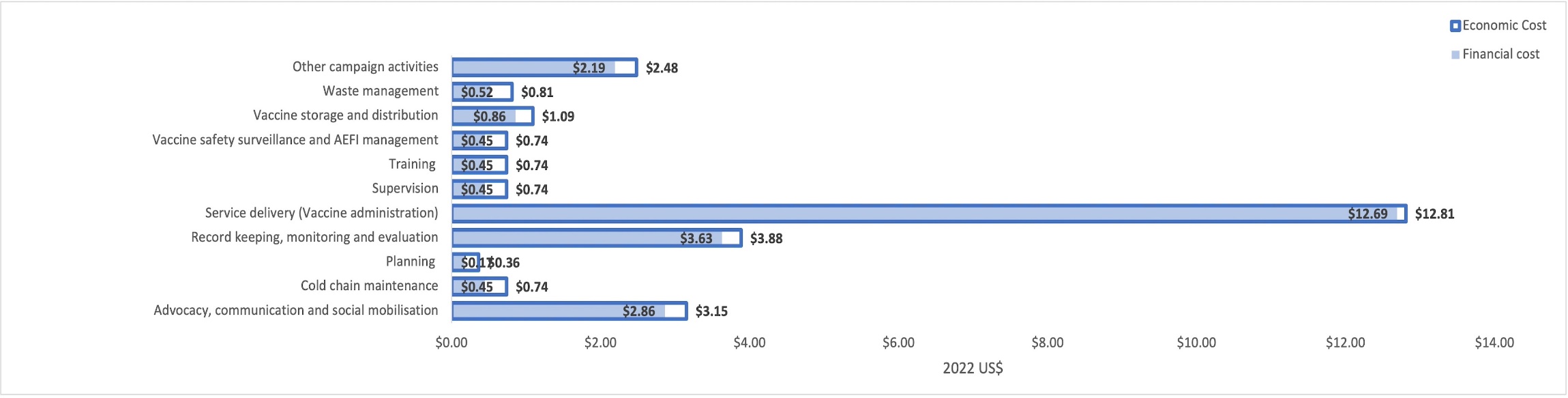


**Tables A5- Total cost (2021 US$) by administrative level and resource type**

| **NATIONAL** |  | | | |
| --- | --- | --- | --- | --- |
|  | **Financial cost (US$)** | **Financial cost (%)** | **Economic Cost (US$)** | **Economic Cost (%)** |
| Vaccine | 6 203 212 | 98% | 6 203 212 | 96% |
| Consumables | 0 | 0% | 0 | 0% |
| Vehicles and transport | 130 832 | 2% | 130 832 | 2% |
| Staff time | 0 | 0% | 97 290 | 2% |
| Per diem | 0 | 0% | 0 | 0% |
| Equipment and supplies | 13 648 | 0.2% | 15 036 | 0.2% |
| Other expenses | 0 | 0% | 0 | 0% |
| **Total** | **6 347 692** | **100%** | **6 446 369** | **100%** |
| **DISTRICT** |  | | | |
|  | **Financial cost (US$)** | **Financial cost (%)** | **Economic Cost (US$)** | **Economic Cost (%)** |
| Vaccine | 0 | 0% | 0 | 0% |
| Consumables | 0 | 0% | 0 | 0% |
| Vehicles and transport | 26 880 | 41% | 26 971 | 9% |
| Staff time | 0 | 0% | 236 659 | 78% |
| Per diem | 18 864 | 29% | 18 864 | 6% |
| Equipment and supplies | 13 119 | 20% | 13 327 | 4% |
| Other expenses | 6 542 | 10% | 6 542 | 2% |
| **Total** | **65 406** | **100%** | **302 364** | **100%** |
| **HOSPITAL** |  | | | |
|  | **Financial cost (US$)** | **Financial cost (%)** | **Economic Cost (US$)** | **Economic Cost (%)** |
| Consumables | 17 117 | 13% | 17 117 | 3% |
| Vehicles and transport | 2 733 | 2% | 2 398 | 0.4% |
| Staff time | 84 136 | 62% | 535 386 | 91% |
| Per diem | 0 | 0% | 0 | 0% |
| Equipment and supplies | 29 891 | 22% | 32 744 | 6% |
| Other expenses | 2 210 | 2% | 2 210 | 0.4% |
| **Total** | **136 087** | **100%** | **589 855** | **100%** |
| **PRIMARY HEALTH CARE** |  | | | |
|  | **Financial cost** | **Financial cost (%)** | **Economic Cost** | **Economic Cost (%)** |
| Consumables | 122 948 | 16% | 122 948 | 7% |
| Vehicles and transport | 0 | 0% | 0 | 0% |
| Staff time | 536 962 | 71% | 1 485 717 | 87% |
| Per diem | 0 | 0% | 0 | 0% |
| Equipment and supplies | 80 410 | 11% | 89 403 | 5% |
| Other expenses | 13 259 | 2% | 13 259 | 1% |
| **Total** | **753 578** | **100%** | **1 711 327** | **100%** |
| **FIXED OUTREACH** |  | | | |
|  | **Financial cost (US$)** | **Financial cost (%)** | **Economic Cost (US$)** | **Economic Cost (%)** |
| Consumables | 35 835 | 4% | 35 835 | 2% |
| Vehicles and transport | 5 929 | 1% | 5 929 | 0.3% |
| Staff time | 225 738 | 27% | 1 609 288 | 71% |
| Per diem | 516 706 | 62% | 516 706 | 23% |
| Equipment and supplies | 46 802 | 6% | 47 640 | 2% |
| Other expenses | 5 893 | 1% | 56 927 | 3% |
| **Total** | **836 903** | **100%** | **2 272 326** | **100%** |
| **TEMPORARY OUTREACH** |  | | | |
|  | **Financial cost (US$)** | **Financial cost (%)** | **Economic Cost (US$)** | **Economic Cost (%)** |
| Consumables | 14 645 | 7% | 14 645 | 2% |
| Vehicles and transport | 20 507 | 9% | 20 547 | 3% |
| Staff time | 6 258 | 3% | 408 101 | 65% |
| Per diem | 158 409 | 73% | 158 409 | 25% |
| Equipment and supplies | 13 939 | 6% | 27 631 | 4% |
| Other expenses | 2 946 | 1% | 2 946 | 0.5% |
| **Total** | **216 705** | **100%** | **632 279** | **100%** |
| **MOBILE OUTREACH** |  | | | |
|  | **Financial cost (US$)** | **Financial cost (%)** | **Economic Cost (US$)** | **Economic Cost (%)** |
| Consumables | 1 355 | 1% | 1 355 | 1% |
| Vehicles and transport | 5 090 | 5% | 5 090 | 5% |
| Staff time | 17 754 | 19% | 29 329 | 27% |
| Per diem | 57 941 | 61% | 57 941 | 54% |
| Equipment and supplies | 13 066 | 14% | 14 390 | 13% |
| Other expenses | 123 | 0.1% | 123 | 0.1% |
| **Total** | **95 328** | **100%** | **108 228** | **100%** |

**Figure A2- Cost per dose (2021 US$) by administrative level and resource type**

*National level*


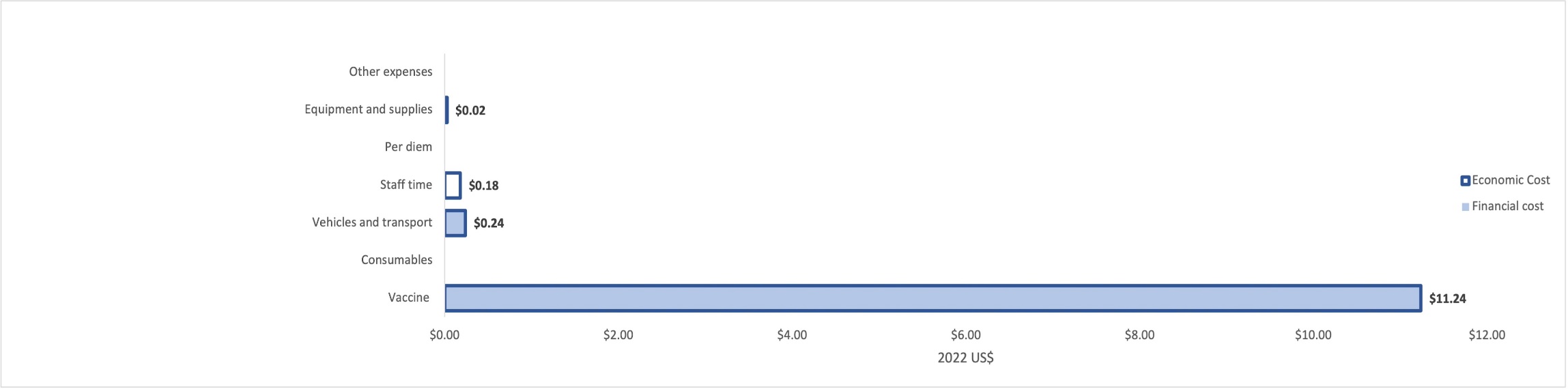


*District level*


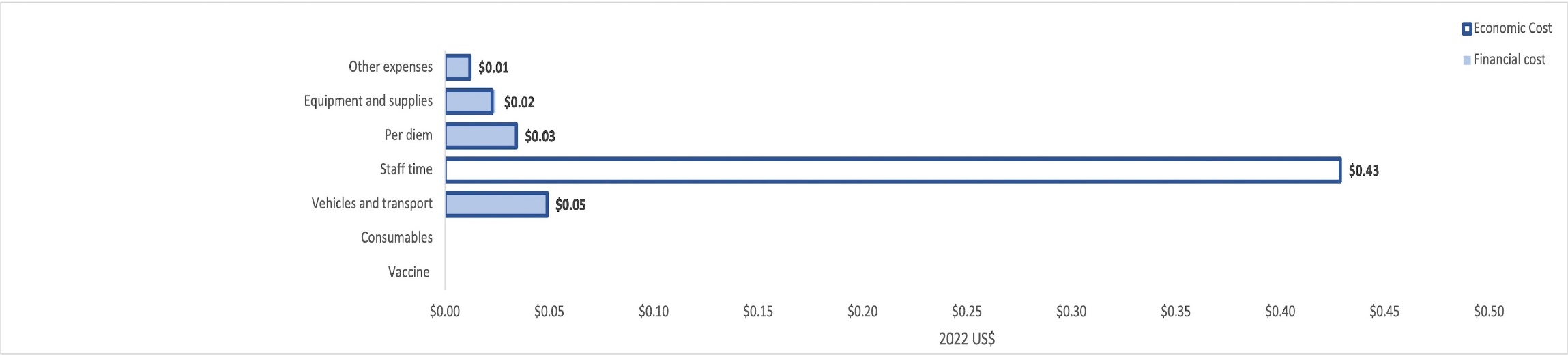


*Hospital level*


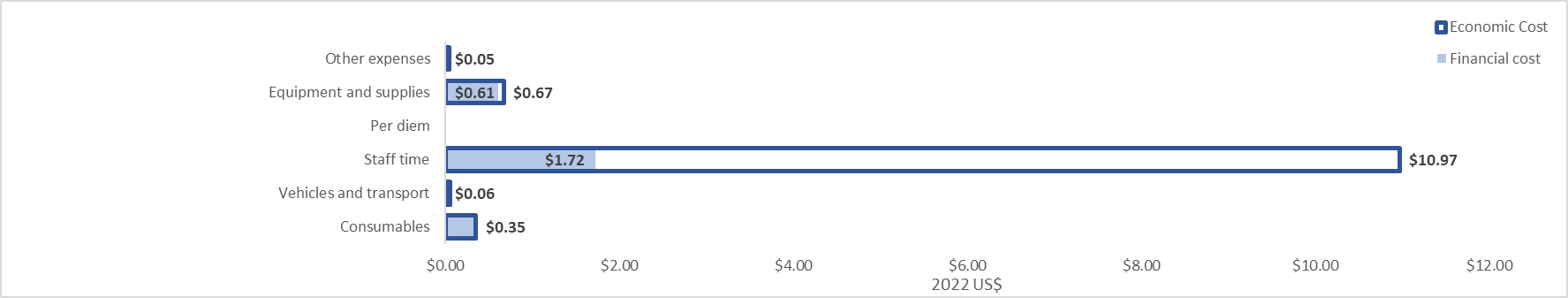


*Primary healthcare level*


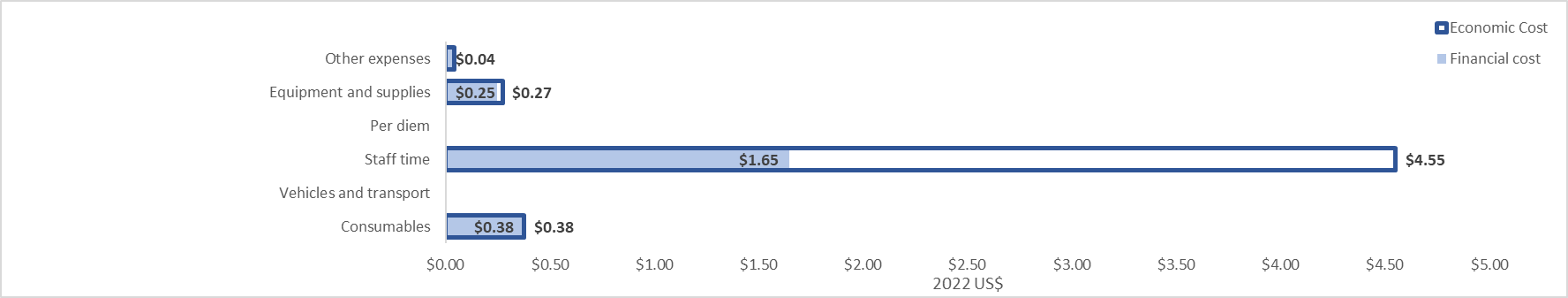


*Fixed outreach level*


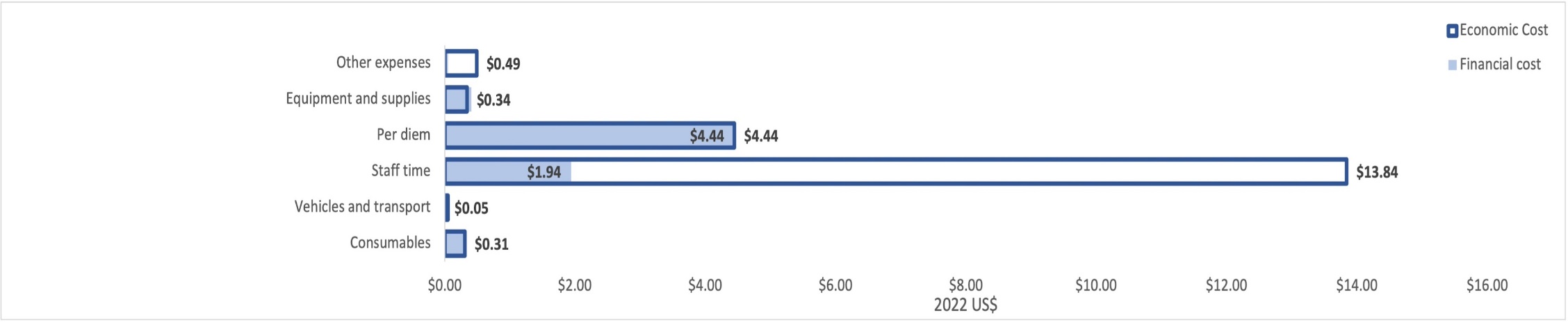


*Temporary outreach level*


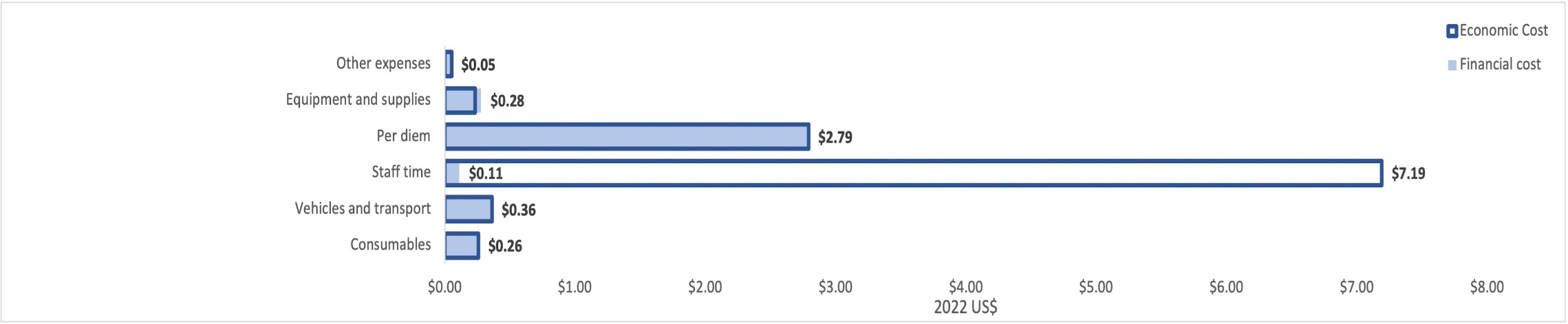


*Mobile outreach*


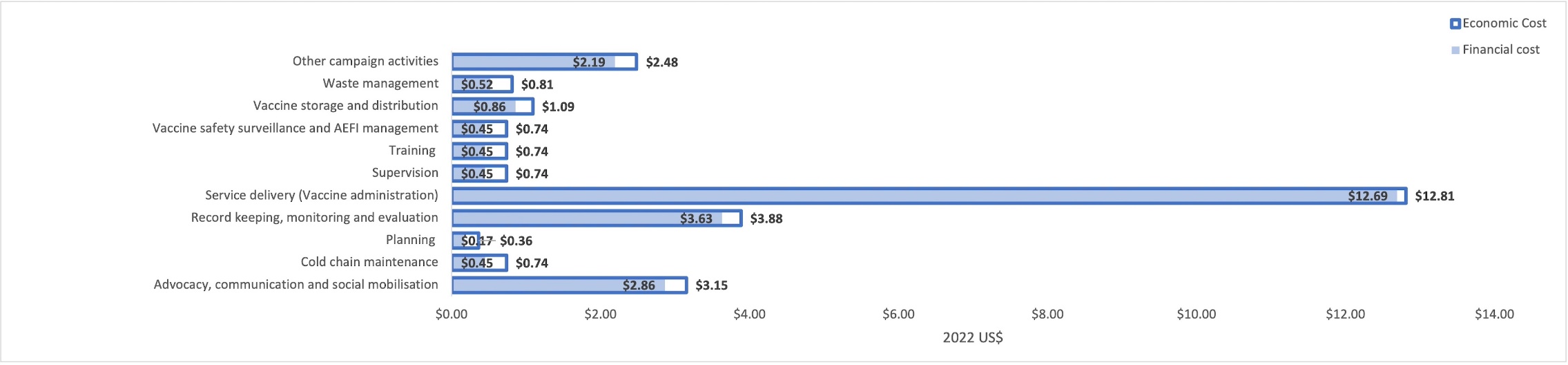


**Table A6: Number of doses administered, wasted doses, and allocation factors , January 2021-January 2022**

| **Administrative level** | **Total**  **#doses** | **Pfizer**  **#doses administered** | **Jansen (J&J)**  **# doses administered** | **Total**  **#Wasted doses** | **National allocation weight** | **District**  **allocation weight** |
| --- | --- | --- | --- | --- | --- | --- |
| National (public sector doses) | 22 410 869 | 15 973 314 | 6 437 555 |  |  |  |
| West Rand District | 552 106 | 453 336 | 98 770 | 22 447 | 2% |  |
| Hospital | 48 804 | 47 769 | 1 035 | 881 | 0.2% | 9% |
| PHC | 326 415 | 271 029 | 55 386 | 21 500 | 1% | 59% |
| Fixed outreach | 116 271 | 99 821 | 16 450 | 6 | 0.4% | 21% |
| Temporary outreach | 56 752 | 31 901 | 24 851 | 26 | 0.2% | 10% |
| Mobile outreach | 3 864 | 2 816 | 1 048 | 34 | 0% | 1% |

**Table A7: Capital vs recurrent cost (2021 US$), January 2021 to January 2022**

|  | **Capital costs** | | **Recurrent cost** | | **Allocation to total cost** | |
| --- | --- | --- | --- | --- | --- | --- |
| **Administrative level** | **Financial (US$)** | **Economic (US$)** | **Financial (US$)** | **Economic (US$)** | **Capital cost (%)** | **Recurrent Cost (%)** |
| National* | 13 648 | 16 786 | 6 087 094 | 6 182 633 | 0.2% | 99.8% |
| District | 8 188 | 59 891 | 57 218 | 242 473 | 19% | 81% |
| Hospital | 7 968 | 42 868 | 137 903 | 556 771 | 7% | 93% |
| PHC | 79 018 | 152 973 | 911 011 | 1 794 805 | 8% | 92% |
| Fixed Outreach | 19 658 | 129 369 | 817 312 | 2 143 024 | 5% | 95% |
| Temporary Outreach | 13 826 | 45 887 | 203 154 | 586 668 | 7% | 93% |
| Mobile Outreach | 2 409 | 8 069 | 93 291 | 100 531 | 5% | 95% |
| **Total** | **144 716** | **455 843** | **8 306 983** | **11 606 904** | **3%** | **97%** |

**Figure A3: Impact of variations in discount rate on total cost (2021 US$) and cost per dose (2021 US$)**

*Total cost (US$)- Discount rate Cost per dose (US$)- Discount rate*

*
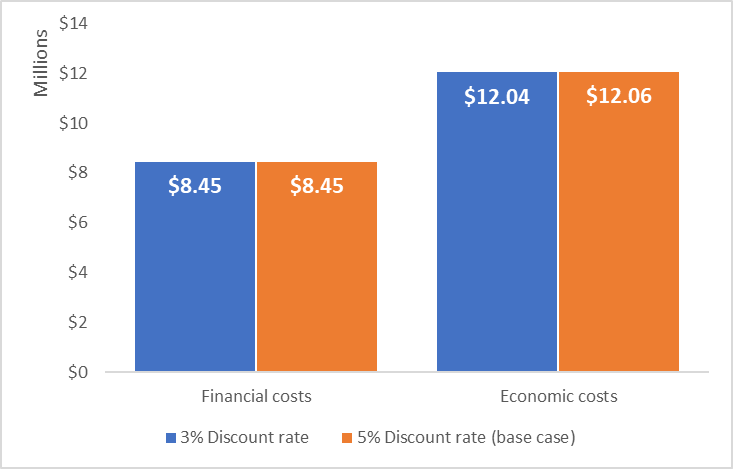

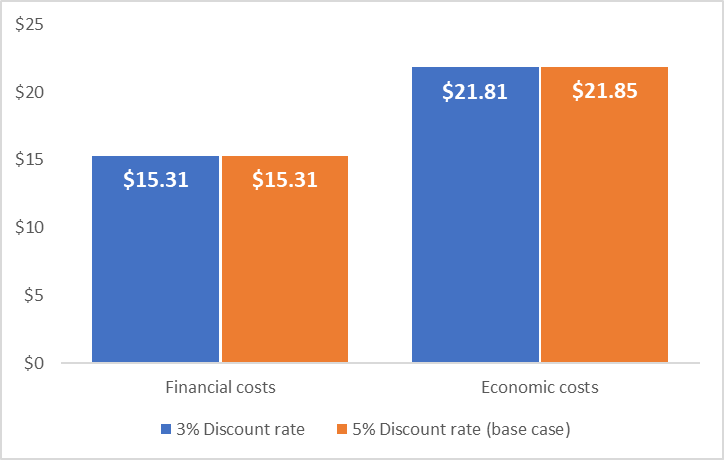
*

**Figure A4: - Impact of variations in useful life on total cost (2021 US$) and cost per dose (2021 US$)**

*Total cost (US$)- Useful years Cost per dose (US$)- Useful years*


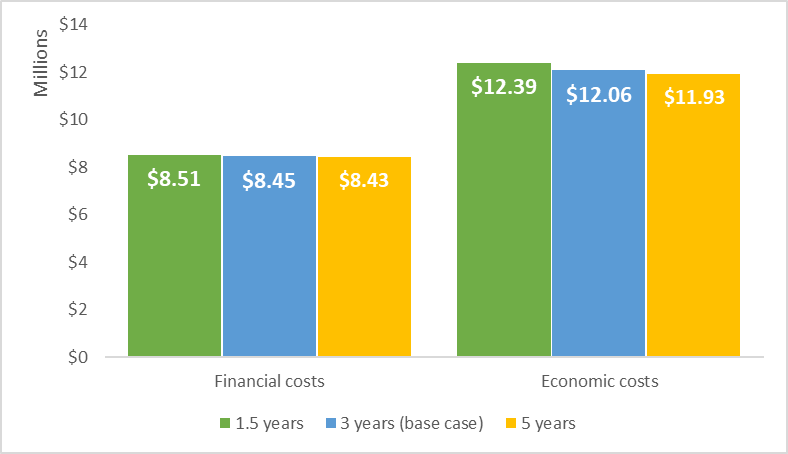

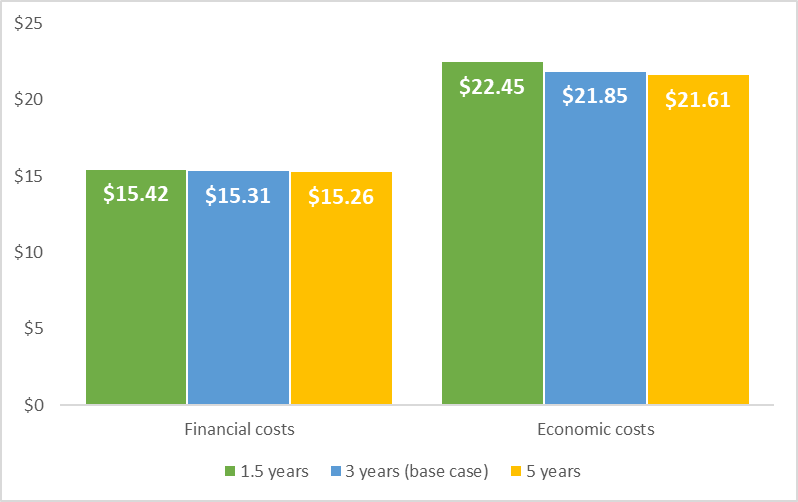


**Figure A5: - Impact of classifying planning programme activity resources as capital vs recurrent costs on total cost (2021 US$) and cost per dose (2021 US$)**

*Total cost (US$)- Planning programme activity resources Cost per dose (US$)- Planning programme activity* *resources*


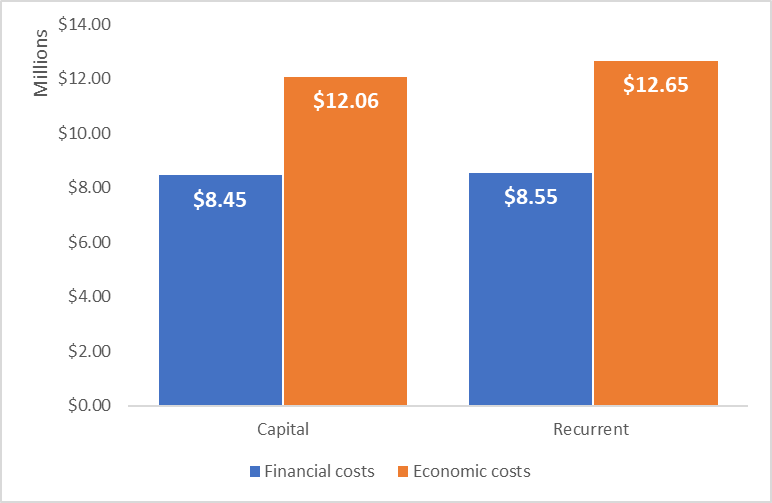

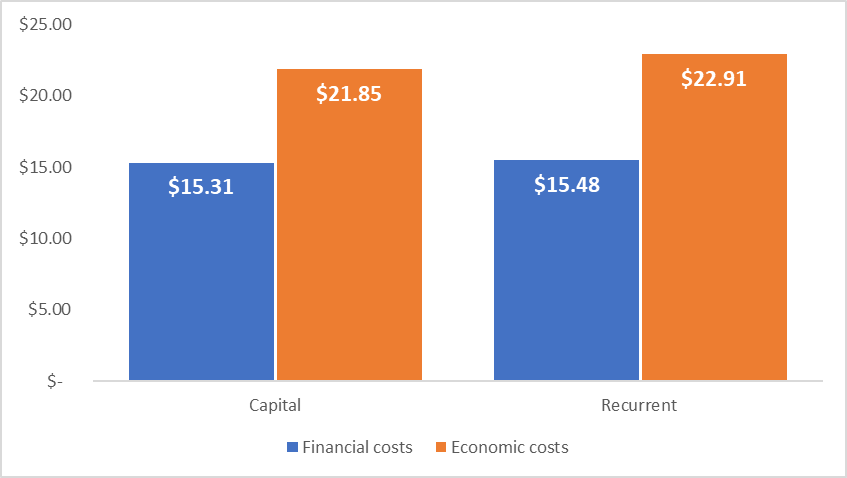


Table A8: CHEERS Checklist

|  | **Item** | **Guidance for Reporting** | **Reported in section** |
| --- | --- | --- | --- |
| **TITLE** | | |  |
| Title | 1 | Identify the study as an economic evaluation and specify the interventions being compared. | Not applicable |
| **ABSTRACT** | | |  |
| Abstract | 2 | Provide a structured summary that highlights context, key methods, results and alternative analyses. | Abstract page 2 |
| **INTRODUCTION** | | |  |
| Background and objectives | 3 | Give the context for the study, the study question and its practical relevance for decision making in policy or practice. | Background, page 3-4 |
| **METHODS** | | |  |
| Health economic  analysis plan | 4 | Indicate whether a health economic analysis plan was developed and  where available. | Methods page 7-14 |
| Study population | 5 | Describe characteristics of the study population (such as age range, demographics, socioeconomic, or clinical characteristics). | Not applicable |
| Setting and location | 6 | Provide relevant contextual information that may influence findings. | Background, page 4 |
| Comparators | 7 | Describe the interventions or strategies being compared and why chosen. | Not applicable |
| Perspective | 8 | State the perspective(s) adopted by the study and why chosen. | Background, page 4 |
| Time horizon | 9 | State the time horizon for the study and why appropriate. | Background, page 4 |
| Discount rate | 10 | Report the discount rate(s) and reason chosen. | Methods, page 14 |
| Selection of outcomes | 11 | Describe what outcomes were used as the measure(s) of benefit(s) and harm(s). | Not applicable |
| Measurement of outcomes | 12 | Describe how outcomes used to capture benefit(s) and harm(s) were measured. | Not applicable |
| Valuation of outcomes | 13 | Describe the population and methods used to measure and value outcomes. | Not applicable |
| Measurement and valuation of resources  and costs | 14 | Describe how costs were valued. | Methods pages 7-14 |
| Currency, price date, and conversion | 15 | Report the dates of the estimated resource quantities and unit costs, plus the currency and year of conversion. | Methods page 7-8 |
| Rationale and  description of model | 16 | If modelling is used, describe in detail and why used. Report if the model  is publicly available and where it can be accessed. | Not applicable |
| Analytics and assumptions | 17 | Describe any methods for analysing or statistically transforming data, any extrapolation methods, and approaches for validating any model used. | Methods, page 12-14 |
| Characterizing heterogeneity | 18 | Describe any methods used for estimating how the results of the study vary for sub-groups. | Not applicable |
| Characterizing  distributional effects | 19 | Describe how impacts are distributed across different individuals  or adjustments made to reflect priority populations. | Not applicable |
| Characterizing uncertainty | 20 | Describe methods to characterize any sources of uncertainty in the analysis. | Method, page 14 |
| Approach to engagement with patients and others affected by the study | 21 | Describe any approaches to engage patients or service recipients, the general public, communities, or stakeholders (e.g., clinicians or payers) in the design of the study. | Not applicable |
| **RESULTS** | | |  |
| Study parameters | 22 | Report all analytic inputs (e.g., values, ranges, references) including uncertainty or distributional assumptions. | Supplemental table A1-A3 |
| Summary of main results | 23 | Report the mean values for the main categories of costs and outcomes of interest and summarise them in the most appropriate overall measure. | Results, Tables 2-4 |
| Effect of uncertainty | 24 | Describe how uncertainty about analytic judgments, inputs, or projections  affect findings. Report the effect of choice of discount rate and time horizon, if applicable. | Results, page 19 |
| Effect of engagement with patients and others affected by the study | 25 | Report on any difference patient/service recipient, general public, community, or stakeholder involvement made to the approach or findings of the study | Not applicable |
| **DISCUSSION** | | |  |
| Study findings, limitations, generalizability, and current knowledge | 26 | Report key findings, limitations, ethical or equity considerations not captured, and how these could impact patients, policy, or practice. | Discussion, page 22-23 |
| **OTHER RELEVANT INFORMATION** | | | |
| Source of funding | 27 | Describe how the study was funded and any role of the funder in the identification, design, conduct, and reporting of the analysis | Funding, page 24 |
| Conflicts of interest | 28 | Report authors conflicts of interest according to journal or  International Committee of Medical Journal Editors requirements. | Competing interest, page 24 |

Husereau D, Drummond M, Augustovski F, de Bekker-Grob E, Briggs AH, Carswell C, Caulley L, Chaiyakunapruk N, Greenberg D, Loder E, Mauskopf J, Mullins CD, Petrou S, Pwu RF, Staniszewska S; CHEERS 2022 ISPOR Good Research Practices Task Force. Consolidated Health Economic Evaluation Reporting Standards 2022 (CHEERS 2022) Statement: Updated Reporting Guidance for Health Economic Evaluations. BMJ. 2022;376:e067975. The checklist is Open Access distributed in accordance with the terms of the Creative Commons Attribution (CC BY 4.0) license, which permits others to distribute, remix, adapt and build upon this work, for commercial use, provided the original work is properly cited. See: http://creativecommons.org/licenses/by/4.0/.
